# Sepsis-related hospital admissions and ambient air pollution: a time series analysis in 6 Chinese cities

**DOI:** 10.1186/s12889-021-11220-x

**Published:** 2021-06-21

**Authors:** Yu Wang, Zhen Liu, Lian Yang, Jiushun Zhou, Jia Li, Hai Lun Liao, Xing Jun Tian

**Affiliations:** 1 Department of Anesthesiology, Union Hospital, Tongji Medical College, Huazhong University of Science and Technology, Wuhan, 430022 China; 2 Second Affiliated Hospital of Chengdu Medical College, China National Nuclear Corporation 416 Hospital, Chengdu, 610057 China; 3 School of Public Health, Chengdu University of Traditional Chinese Medicine, Chengdu, 610075 China; 4 Sichuan Center for Disease Control and Prevention, Chengdu, 610041 China; 5 Management College, Chengdu University of Traditional Chinese Medicine, Chengdu, 610032 China; 6 Sichuan Administration of TCM, Chengdu, 610016 China

**Keywords:** Sepsis, Air pollution, Hospital admission, Time series, China

## Abstract

**Background:**

Some prevalent but rarely studied causes of hospital admissions, such as sepsis is still unknown whether affected by air pollution.

**Methods:**

We used time-series regression within generalized additive models to estimate the effect of air pollutant level on the sepsis-related hospital admissions, for the years 2017–18, using data from six cities in Sichuan, China. Potential effect modifications by age and sex were also explored. The effects of air pollutant on hospital stays for sepsis were also quantified.

**Results:**

Positive associations between short-term exposure to NO_2_ and O_3_ and risk of sepsis-related hospital admissions and stays were found. Each 10 μg/m^3^ increase in short-term NO_2_ at lag 03 and O_3_ at lag 4 was associated with an increase of 2.76% (95% CI: 0.67, 4.84%) and 0.64% (95% CI: 0.14, 1.14%) hospital admissions, respectively. An increase of 0.72% (95% CI: 0.05, 1.40%) hospital stay was associated with 10 μg/m^3^ increase in O_3_ concentration at lag 4. Besides, the adverse effect of exposure to NO_2_ was more significant in males and population aged less than 14 years; while more significant in females and population aged 14 ~ 65 and over 65 years for exposure to O_3_. These associations remained stable after the adjustment of other air pollutants.8.

**Conclusion:**

Exposure to ambient NO_2_ and O_3_ may cause substantial sepsis hospitalizations, and hospital stays in Sichuan, China. These associations were different in subgroup by age and sex.

**Supplementary Information:**

The online version contains supplementary material available at 10.1186/s12889-021-11220-x.

## Background

During the past two decades, the body of evidence regarding the hazardous effect of ambient air pollution on public health has grown substantially, including particulate matter with aerodynamic diameter ≤ 2.5 μm (PM_2.5_) and ≤ 10 μm (PM_10_), nitrogen dioxide (NO_2_), ozone (O_3_) and sulphur dioxide (SO_2_) and carbon monoxide (CO). Our study design was informed by the strong association between air pollution, and multi-organ diseases and injury, including cardio-respiratory disease, stroke, genitourinary diseases, and gastrointestinal diseases [1–3].

Sepsis is a severe syndrome of systemic inflammatory response with a proven or suspected infectious etiology. Severe sepsis is associated with organ dysfunction distant from the site of infection, and may be accompanied by shock. It could be triggered by microbial infection such as pneumonia, kidney infection, cellulitis, and meningitis [4]. In 2017, 48 million cases of sepsis were recorded globally, including 11 million sepsis related deaths, accounting for 19.7% of all deaths worldwide [5]. A recent study conducted by Christopher, et al. demonstrated that higher level of PM_2.5_ may increase the intensive care unit (ICU) mortality in patients with sepsis [6]. They believed that larger studies are required to determine if the frequency of ICU admissions is positively associated with short-term exposure to air pollution. The impact of chronic exposure to air pollution on sepsis-related mortality was explored among 444,928 patient who met the Angus definition of sepsis [7]. In this study, O_3_ but not PM_2.5_ air pollution was associated with higher risk of mortality in patients with sepsis, which is not consistent with Christopher’s findings. A nested case-control study based on an ongoing national longitudinal cohort, evaluated the effect of PM_2.5_ on risk of sepsis hospitalization [8]. The results showed that PM_2.5_ air pollution exposure was not associated with risk of sepsis.

While multiple air pollutants has been associated with health complications, studies evaluating the impact of chronic exposure to air pollution on sepsis is still mixed and limited. To our knowledge no prior study has evaluated the associations between sepsis hospitalization and air pollutants except O_3_ and PM_2.5_. Besides, there has been no systematic research on the effect of multi-urban agglomeration and multi-pollutant on the sepsis. All of these point together towards a need for a comprehensive understanding of the effects on sepsis induced by short-term air pollution exposure.

In southwest China, nearby Sichuan basin and the hengduan mountains, the Sichuan region is one of China’s most polluted region. It may be caused by the basin and the surrounding plateau alpine terrain, atmospheric pollutant dispersion degree is relatively slow. Though the total area in Sichuan region account for only 2.7% in China, the PM_2.5_, NO_2_, SO_2_ emissions occupy respectively accounted for 8.3, 12.1, 5.8% of the whole of China [9, 10]. Nearly 100 million in Sichuan region may suffer from air pollutants. Thus, the purpose of this study is to evaluate the impact of short-term exposure to air pollutants (PM_2.5_, PM_10_, SO_2_, NO_2_, and O_3_) on sepsis hospitalizations in 6 cities of Sichuan region from January 1, 2017 to January 1, 2019.

## Methods

### Study setting

**Fig. 1:**
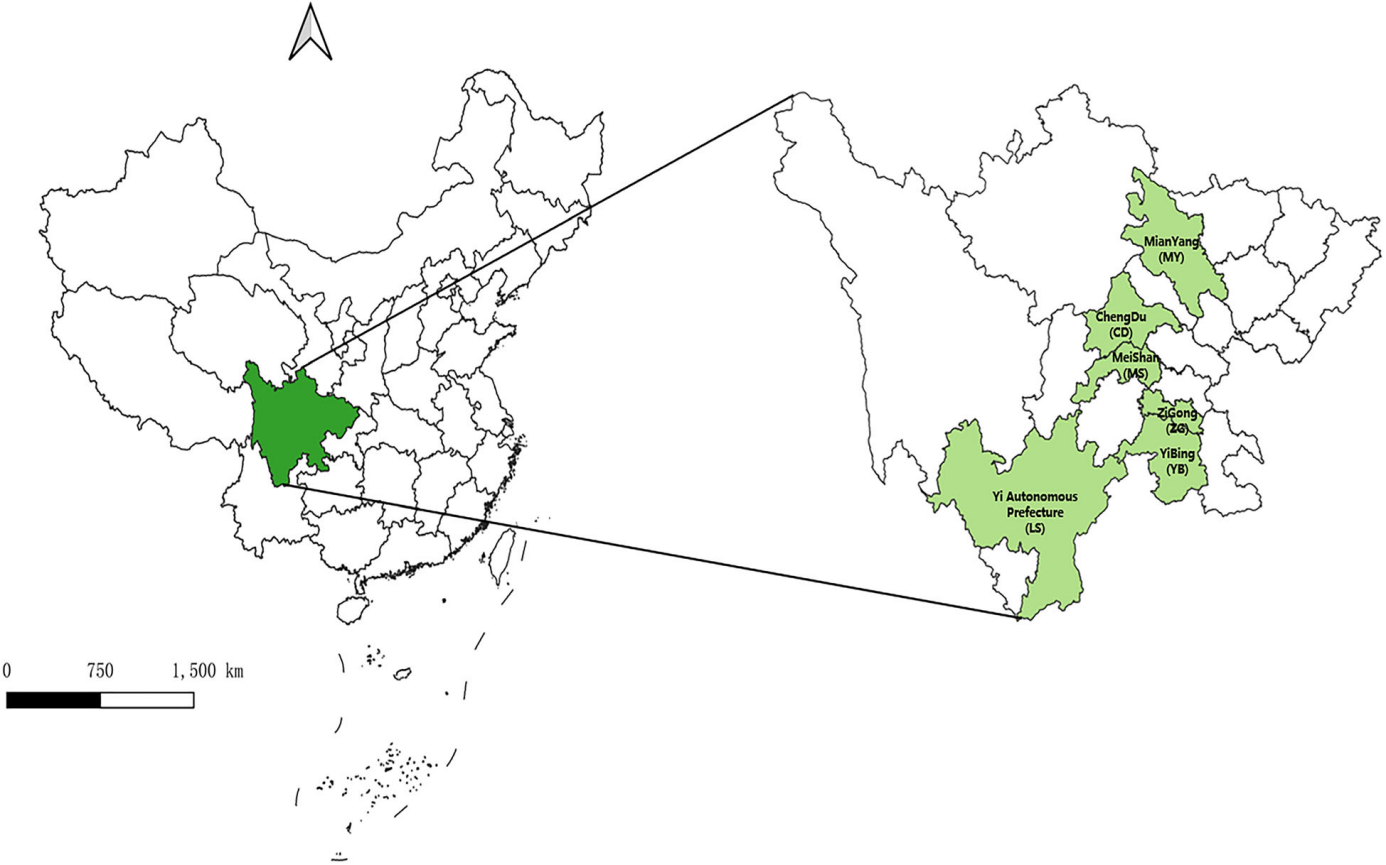
The geographical distribution of the 6 cities in Sichuan, China. The location of study cities in Sichuan region were marked in green

A multi-stage stratified cluster sampling method was used to extract samples from medical and health institutions. In the first stage, according to the sampling principles such as the level of economic and health services, and geographical location the Sichuan region, six cities were selected in our study. Cities of plain areas include Chengdu (CD), Meishan (MS), hilly areas include Mianyang (MY), Yibin (YB), Zigong (ZG), and plateau areas include Yi Autonomous Prefecture (LS). The geographical distributions of the six cities in Sichuan, China were displayed in Fig. 1.

In the second stage, 4 counties (districts) are selected from each city as the sample counties (districts). In the third stage, general hospitals, traditional Chinese medicine hospitals, specialized hospitals, maternity and child care hospitals and some primary medical and health institutions will be selected from each sample county (district). Records of the electronic medical information system were extracted from all the medical health institutions in the 6 cities. A total of 412 medical and health institutions were involved. The population covered in this study exceeded 35 million, and the proportion of inpatients included accounted for more than 80% of the inpatients in all medical and health institutions of the studied cities.

### Environmental data

Ambient air quality data were collected from the China National Environmental Monitoring Center and Department of Ecology and Environment of Sichuan Province. Hourly concentrations of PM_10_, PM_2.5_, NO_2_, SO_2_, O_3_, and CO were measured from 67 air monitoring stations interspersed in the 6 cities: 19 in CD, 6 in MS, 9 in MY, 10 in YB, 6 in ZG, and 17 in LS. The daily 24-h mean concentration of air pollution was simply averaged of all valid sites in each city for analyses. To adjust the impact of weather conditions, meteorological data including daily mean temperature and relative humidity were obtained from the Department of Ecology and Environment of Sichuan Province.

### Hospital admissions data

We diagnosed sepsis as the life-threatening organ dysfunction caused by dysregulated response to infection, and only required suspected infection rather than proven one [11]. Overall sepsis estimates were based on both explicit and implicit with organ dysfunction codes, according to the approach used in epidemiology studies of sepsis [5, 12]. The cases diagnosed as both International Classification of Diseases 10th and 9th Revision Code (ICD-10 and ICD-9) referencing sepsis explicitly were explicit sepsis; cases could not meet criteria for explicit sepsis but with the code of organ dysfunction was eligible to be classified as implicit sepsis with organ dysfunction group. Detailed ICD-10 and ICD-9 codes for the identification of explicit sepsis and implicit sepsis were listed in the Table S1.

Some patients suffered the sepsis during the period of the hospitalization. We only collected the sepsis patients with the primary admission diagnosis, which were more vulnerable to air pollutants before admissions. The sepsis data was obtained from the electronic medical information system of 412 medical and health institutions. After desensitization, the basic characteristics of the patients and the information of medical treatment were obtained.

### Statistical analysis

The original data manipulation was performed using SAS version 10.4, to obtain the daily hospital admissions, daily-average air pollutant concentration and meteorological level. To avoid the bias, and provided the most realistic daily air pollutant exposure on population, residential postcode of patients was used to match sepsis episodes and link with air pollution data, rather than the hospital postcode. Then we applied a two-stage analytical approach to estimate the city-specific and multi-cities average associations between daily air pollution levels and sepsis-related hospital admissions.

In the first stage, since daily hospital admissions approximately followed a quasi-Poisson distribution, we used generalized additive models (GAM) to estimate the city-specific short-term effects of air pollution. Several covariates were adjusted in the main models as a standardized protocol in time-series analyses [13–15]: 1) a natural spline function of calendar time with 7 degrees of freedom (df) per year to account for potential temporal trends in hospitalizations; 2) a dummy variable for days of week to eliminate the weekday effect; 3) a natural spline function of present-day average temperature and relative humidity with respective 6 dfs and 3 dfs to account for potential confounding effects of weather factors [13–15]. Briefly, the main model is as follows: *log*[*E*(*Y*_*t*_)] = α + *DOW* + Z_*t*_ + *ns*(*day*, *df*) + *ns*(*temperature*, *df*) + *ns*(*relative humidity*), where E (Y_t_) indicates the estimated number of hospital admissions for sepsis on day t; Z_t_ denotes the air pollutant concentrations on day t; ns is a natural spline function; df is the degrees of freedom; DOW is the dummy variable for day of the week, and α is the intercept.

In the second stage, random-effects meta-analyses were applied to combine the city-specific estimates to pool the average estimates at a regional level [16, 17]. To search the lag patterns in the impacts of air pollutants on sepsis, we further introduced both single-day lags and moving average lag days, using the same models. Single-day lag structures (lag 0 to lag 7) were the days before the hospital admissions. For example, lag 0 is the current day of hospital admission; lag 1 and lag 2 is the 1 day before admission and 2 days before admission. The moving average lag days were cumulative lag effects of air pollutants effects from lag 01 to lag 06 days. For example, lag 01 was the 2-day moving average concentration computed as the means of lag 0 and lag 1 days; lag 06 was the 7-day moving average concentration computed as the means of from lag 0 to lag 6 days.

We further conducted stratified analyses by sex, and age groups (0 ~ 13 years; 14 ~ 65 years; > 65 years) to explore the potential effect modifications among different population. Sensitivity analyses were conducted to assess the stability and robustness of the estimates in two-pollutant models. Two-pollutant models aimed to adjust the other air pollutants using the same parameter settings as in the main mode. Furthermore, we also analyzed the association between the hospital stay of sepsis and short-term exposure to air pollutants which had significant effects on hospitalizations. Since days of hospitalization displayed a similar distribution with daily hospitalizations, the same model setting was used to fit associations of air pollution with hospital stays [18].

All analyses were conducted in R version 3.4.1 (R Foundation for Statistical Computing, Vienna, Austria), with the “mgcv” package and “rmeta” package. The estimates are reported as percentage changes and 95% confidence intervals (95% CI) in daily sepsis-related hospital admissions associated with per 10 μg/m^3^ increase in air pollutant concentrations. A two-sided alpha level of 0.05 (*P*-value< 0.05) was considered statistically significant.

## Results

### Descriptive results

From the medical records from 6 large cities in Sichuan, China, 58,064 hospital admissions for sepsis occurred from 1st January 2017 to 31st December 2018 (731 days). Overall, there were 57.4% female patients and 49.8% patients aged 14 ~ 65 years. The daily average values of hospital stays and hospitalizations for sepsis were 119 days and 13 cases, respectively. Daily concentrations of air pollutants were comparatively low during the study period, with a daily average of 41.4, 63.8, 12.1, 25.3, 82.7 μg/m^3^ for PM_2.5_, PM_10_, NO_2_, SO_2_, O_3_, and 0.8 mg/m^3^ for CO, compared with the air quality guideline for limit of pollutant concentration in China (Table S2). The daily average temperature and relative humidity were 17.5 °C and 76.3%, respectively. More detailed information for these descriptive statistics with sepsis can be found in Table 1.

**Table 1:** Summary statistics of data on hospital admissions and stays of sepsis, air pollutants, and weather conditions in 6 Sichuan cities, 2017–18

	Number (%)	Mean	SD	MIN	Median	MAX	Range
Hospital admissions of sepsis							
total	58,064	13	9	7	11	30	23
0 ~ 13 years	16,325 (28.1%)	4	3	1	3	9	7
14 ~ 65 years	28,905 (49.8%)	7	4	4	5	14	10
> 65 years	12,834 (22.1%)	3	2	0	3	8	7
male	24,764 (42.6%)	6	4	3	5	13	10
female	33,300 (57.4%)	8	5	4	6	17	12
Hospital stays of sepsis		119	88	57	91	288	231
24-h average air pollutant concentrations, μg/m^3^							
PM_2.5_		41.4	13.2	17.6	45.0	54.9	37.3
PM_10_		63.8	15.9	35.0	67.4	77.8	42.8
NO_2_		12.1	3.6	8.2	11.1	18.4	10.2
SO_2_		25.3	7.8	12.9	25.9	36.8	23.8
O_3_		82.7	7.9	74.1	82.8	93.0	18.9
CO		0.8	0.1	0.6	0.8	0.9	0.3
24-h average weather conditions							
temperature		17.5	1.2	15.5	17.6	18.8	3.4
relative humidity		76.3	5.2	66.9	78.0	81.7	14.9

Abbreviations: *SD* standard deviation; *MIN* minimal; *MAX* maximal

### GAM results

Table 2 presented the percentage change of sepsis hospitalizations associated with the increases of different air pollutants level. We found that NO_2_-sepsis associations were significant at the current day (lag 0), lag 1, and lag 01 to lag 04, with the largest estimates of 2.76% (95% CI: 0.67, 4.84%) at lag 03. The increase of O_3_ concentration were also statistically significant related with the sepsis hospitalizations at lag 3, lag 4 and lag 7, and the largest estimates was 0.64% (95% CI: 0.14, 1.14%) at lag 4. The city-specific associations between NO_2_, O_3_ and sepsis-related hospital admissions have been supplemented in the Table S3.

**Table 2:** Percentage increase (estimates and 95% CI) in daily hospital admissions of sepsis associated with 10-μg/m^3^ increase in PM_10_, PM_2.5_, SO_2_, NO_2_, O_3_ and 1 mg/m^3^ CO concentrations in different lag days and moving average days in 6 Sichuan cities

Lag days	PM_10_	PM_2.5_	SO_2_	NO_2_	O_3_	CO
lag0	−0.04(− 0.57, 0.48)	−0.33(− 1.15, 0.49)	1.69(−5.52, 8.89)	**2.54 (0.24, 4.85)**	− 0.62(− 1.25, 0.01)	7.25(− 1.24, 15.70)
lag1	− 0.08(− 0.51, 0.35)	− 0.13(− 1.00, 0.74)	−0.05(−6.65, 6.54)	**1.66 (0.11, 3.21)**	− 0.18(− 0.61, 0.25)	6.43(− 1.85, 14.70)
lag2	− 0.19(− 0.60, 0.22)	− 0.16(− 0.99, 0.68)	0.64(−4.10, 5.39)	1.29(− 0.24, 2.82)	0.06(− 0.32, 0.45)	6.32(−3.99, 16.60)
lag3	− 0.03(− 0.52, 0.46)	−0.07(− 0.91, 0.77)	0.43(− 3.47, 4.34)	0.77(− 0.73, 2.27)	**0.44 (0.07, 0.82)**	6.05(− 1.66, 13.80)
lag4	−0.09(− 0.43, 0.25)	−0.22(− 0.79, 0.35)	0.57(− 2.80, 3.93)	−0.04(− 1.51, 1.43)	**0.64 (0.14, 1.14)**^**a**^	3.23(− 5.40, 11.90)
lag5	− 0.16(− 0.49, 0.18)	−0.40(− 0.86, 0.07)	1.06(− 2.28, 4.41)	0.00(− 1.46, 1.46)	0.17(− 0.19, 0.54)	− 2.49(−9.37, 4.40)
lag6	0.06(− 0.37, 0.48)	−0.06(− 0.56, 0.44)	2.20(− 1.78, 6.17)	−0.17(− 1.97, 1.63)	0.27(− 0.16, 0.70)	0.12(− 8.43, 8.66)
lag7	0.11(− 0.22, 0.45)	−0.01(− 0.46, 0.45)	1.43(− 3.09, 5.94)	− 1.00(− 2.78, 0.78)	**0.48 (0.02, 0.94)**	1.27(− 7.07, 9.60)
lag01	− 0.08(− 0.66, 0.49)	− 0.32(− 1.31, 0.68)	0.70(− 7.84, 9.23)	**2.41 (0.68, 4.15)**	− 0.54(− 1.15, 0.08)	8.66(− 0.90, 18.20)
lag02	− 0.17(− 0.70, 0.36)	− 0.34(− 1.44, 0.76)	0.91(− 8.31, 10.10)	**2.66 (0.74, 4.57)**	− 0.31(− 0.93, 0.32)	11.00(− 1.27, 23.30)
lag03	− 0.15(− 0.76, 0.46)	−0.33(− 1.63, 0.96)	0.85(− 8.60, 10.30)	**2.76 (0.67, 4.84)**^**a**^	0.09(− 0.55, 0.73)	13.70(− 0.18, 27.50)
lag04	− 0.16(− 0.85, 0.52)	−0.41(− 1.88, 1.05)	1.59(− 7.25, 10.40)	**2.51 (0.26, 4.75)**	0.41(− 0.26, 1.07)	14.20(− 1.93, 30.40)
lag05	− 0.29(− 0.89, 0.32)	−0.62(− 1.98, 0.75)	1.98(− 7.28, 11.20)	2.28(− 0.12, 4.69)	0.48(− 0.21, 1.18)	10.90(− 5.40, 27.20)
lag06	−0.28(− 0.95, 0.39)	−0.67(− 2.07, 0.73)	2.91(− 6.18, 12.00)	2.03(− 0.53, 4.59)	0.60(− 0.12, 1.32)	9.50(− 8.52, 27.50)

Note: Statistically significant estimates were marked in bold; ^a^the largest estimate in all lag period

There was also statistically significant association between O_3_ level and sepsis hospital stay at the lag 3, lag 4 and lag 7 (Table 3). An increase of 10 μg/m^3^ of O_3_ at lag 04 was associated with 0.72% (95% CI: 0.05, 1.40%) increase in hospital stay for sepsis. However, we didn’t observe any statistically significant associations between NO_2_ and hospital stay for sepsis.

**Table 3:** Percentage increase (estimates and 95% CI) in hospital stay associated with10-μg/m^3^ increase in NO_2_ and O_3_ concentrations in different lag days and moving average days in 6 Sichuan cities

lag days	NO_2_	O_3_
lag0	0.73(−3.80, 5.27)	−0.71(− 1.69, 0.27)
lag1	0.21(− 1.98, 2.39)	− 0.42(− 1.03, 0.19)
lag2	− 0.44(− 2.60, 1.72)	0.13(− 0.57, 0.82)
lag3	− 0.93(− 3.20, 1.34)	**0.67 (0.05, 1.29)**
lag4	− 1.74(− 3.80, 0.32)	**0.72 (0.05, 1.40)**^**a**^
lag5	0.07(−1.98, 2.12)	0.17(− 0.35, 0.68)
lag6	0.51(−1.52, 2.53)	0.37(− 0.14, 0.88)
lag7	0.21(−2.00, 2.42)	**0.73 (0.22, 1.23)**
lag01	0.05(−4.14, 4.23)	−0.79(−1.68, 0.09)
lag02	0.04(−3.63, 3.71)	−0.51(−1.40, 0.39)
lag03	−0.04(−3.63, 3.54)	0.15(− 0.88, 1.17)
lag04	− 0.68(− 3.82, 2.47)	0.60(− 0.55, 1.75)
lag05	− 0.74(− 4.10, 2.62)	0.65(− 0.47, 1.77)
lag06	−0.79(− 4.36, 2.78)	0.70(− 0.32, 1.72)

Note: Statistically significant estimates were marked in bold; ^a^the largest estimate in all lag period

### Stratified and sensitive analyses

**Fig. 2:**
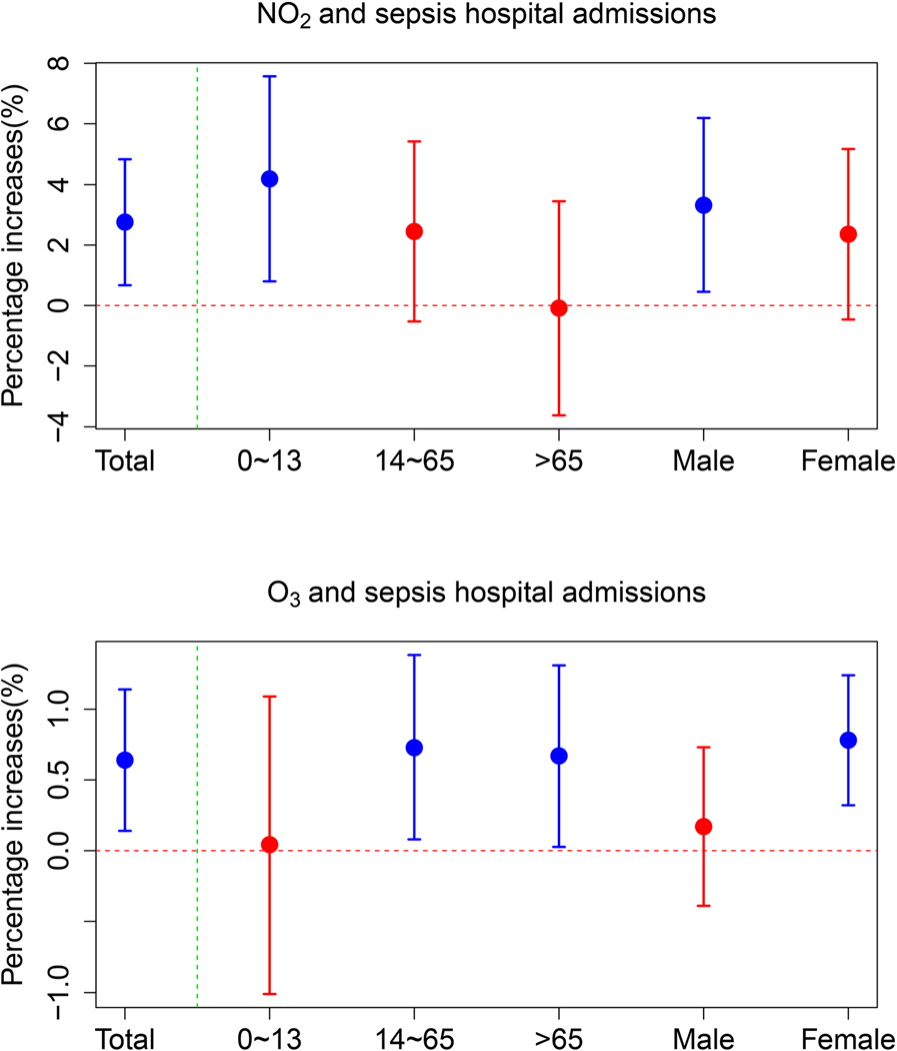
Percentage increase (estimates and 95% CI) in daily hospital admissions of sepsis associated sepsis of 10-μg/m^3^ increase in NO_2_ and O_3_ concentrations at lag days with the highest estimates of basic models for total populations, stratified by age and sex

As the GAM results showed that only NO_2_- and O_3_- sepsis associations were significant at the current day and lag days, the results of stratified analyses and effects on the hospital stay of sepsis were displayed only for NO_2_ and O_3_. The stratified analyses of other air pollutants were supplemented in the Table S4. Multi-cities estimates of the associations between hospital admissions for sepsis and the two air pollutants (NO_2_ and O_3_) stratified by age and sex were displayed **in** Fig. 2. We conducted stratified and sensitive analyses at the lag days which generated the highest estimates in basic models for total populations.

The percentage increases of hospital admissions for sepsis associated with NO_2_, were statistically significant and positive for males and people aged 0 ~ 13 years. While for O_3_, the estimated associations were statistically significant for females, people aged 14 ~ 65 and > 65 years.

Table 4 presented the results of the two-pollutant models to exclude the confounding effects by other air pollutants. The effect estimates remained even unchanged when we adjusted the co-pollutants in the models for O_3_. Besides, the estimated effect of NO_2_ were strengthened, especially with particulate matter adjustments. The NO_2_-related percentage increases for sepsis hospitalizations at lag 03 days were 5.03% (95% CI: 1.42, 8.65) and 4.41% (95% CI: 1.98, 6.84) in two-pollutant models adjusted for PM_10_ and PM_2.5_, respectively.

**Table 4:** Percentage increase (estimates and 95% CI) in daily hospital admissions of sepsis associated sepsis of 10-μg/m^3^ increase in NO_2_ and O_3_ concentrations in lag days with largest estimates, with adjustment of different co-pollutants

	NO_2_	O_3_
Single air pollutant model	2.76 (0.67, 4.84)	0.64 (0.14, 1.14)
Adjusted for PM_2.5_	5.03 (1.42, 8.65)	0.65 (0.13, 1.16)
Adjusted for PM_10_	4.41 (1.98, 6.84)	0.64 (0.14, 1.15)
Adjusted for CO	2.58 (0.30, 4.86)	0.67 (0.16, 1.18)
Adjusted for SO_2_	2.72 (1.07, 4.37)	0.65 (0.18, 1.13)
Adjusted for O_3_ or NO_2_	3.20 (1.08, 5.32)	0.49 (0.21, 0.76)

## Discussion

This study assessed the relationship between sepsis hospital admissions and stays and short-term exposure to six main air pollutants at a multi-city level. We found that increased sepsis-related hospital admission and stay were associated with short-term exposure to NO_2_ and O_3_. The lag patterns which generated the most pronounced effect of NO_2_ and O_3_ differed. Risks of hospital admission for sepsis per NO_2_ and O_3_ concentration increase were different in subgroups by age and sex. Our results support the hypothesis that the sepsis population is affected by air pollutants and warrants further study.

In this study, we estimated percentage increases in hospital admissions and stays for sepsis associated with increase in each air pollutant concentration using a time-series analysis at a 6 large cities level. This time-series analysis has been developed and wildly used to explore the harmful health effect induced by air pollutants on mortality or disease-specific hospital admissions and visits of various diseases [19, 20]. They may increase the all-cause or non-infectious cardiovascular, and respiratory diseases mortality [17, 21] and hospital admissions/visits [14–16]. New issues of the time-series regression model for infectious disease were also hot and developed, including tuberculosis [22], pneumonia [23, 24], lower respiratory infections [25], inflammatory dermatoses [26]. Little is known about the effect of different air pollution on sepsis hospitalizations specifically from the existing researches. We therefore analysed sepsis based on its high prevalence, limited knowledge, shared biological pathways and nascent associations with pollution [7]. Since air pollution and healthcare resources are distributed unevenly across China and world, it may restrict the validity and generalizability of this study. Nevertheless, these 6 cities from Sichuan region in our study were typical cities in Southwest China, and our study could provide the evidence that air pollution may increase the burden of disease and cost from sepsis.

In our study, the effect estimate on sepsis hospital admission was a 0.64% increase per 10 μg/m^3^ increase in O_3_ within 4 days, and 2.76% increase per 10 μg/m^3^ increase in NO_2_ at lag 03. The strongest effect of air pollution presented a time lag with regard to air pollution concentrations, while there was also a suggestion for a cumulative effect. Recently, a time stratified, case crossover analyses conducted in the United States found that short-term exposure to PM_2.5_ is associated with increased risk of hospital admissions due to septicaemia. Absolute increase in risk of admission to hospital of sepsis per 10 million person days associated with per 1 μg/m ^3^ increase PM_2.5_ at lag 01 days was 0.41 (95% CI: 0.29, 0.54) [27]. A number of explanations are possible for the difference between our associations and theirs. Their study population is limited to Medicare population without younger populations, while younger patients (0 ~ 13 years) suffered sepsis accounted almost one-third in our study, which cannot be ignored. On the other hand, the study period was from 2000 to 2012 in their study, and we observed the data in recent years. A recent study stated that point estimates for PM_2.5_ and ICU admission were positive, suggesting a potential association exists, but they failed to observed it as statistically significant [6]. We observed all sepsis patients throughout the hospital, besides the ICU, with a larger sample size (58,064 sepsis cases versus 10,725 sepsis ICU cases [6]). Another study with 444,928 patients of sepsis also reported that exposure to increased levels of O_3_ was associated with higher risk of mortality in patients with sepsis [7], while PM_2.5_ was not associated with sepsis which is similar with our results. For the impact of air pollutants on sepsis hospitalization, a nested case-control study also showed no relationship between PM_2.5_ air pollution and sepsis hospitalization [8]. Therefore, the current evidence of the effect of PM_2.5_ air pollution on sepsis is insufficient and controversial. In addition to the research on particulate matter, studies on gaseous pollutants (SO_2_, NO_2_, O_3_ and CO) and sepsis is more scarce. Our results can provide relevant evidence, while need more research results to compare and verify.

From the results in our study, increased NO_2_ and O_3_ concentration may increase the number of sepsis cases and prolong the duration of hospitalization. However, sepsis is a prevalent but rarely studied causes of hospital admissions that was identified significant associations with short-term exposure to air pollution. Therefore, the physiological processes affected by the ambient air pollutants for sepsis is unknown. Short-term exposure to pollution has been proved to be associated with multiple infectious diseases, including pneumonia [23, 24], lower respiratory infections [25], inflammatory dermatoses [26] in general hospital populations. Sepsis is a critical syndrome of systemic inflammation triggered by infectious diseases and widespread organ dysfunction. Therefore, the risk of sepsis may increase by various infectious diseases which are associated with the air pollution exposure. The association between air pollution and sepsis is likely to come from the effects of air pollution on infectious diseases, or the combined effect of air pollution and infectious diseases. The pathophysiology may overlap with the damage by short-term exposure of air pollution, and the occurrence of infectious diseases. Therefore, we need to realize that air pollution may increase the incidence of sepsis, but it is not a direct pathogenic factor. The corresponding hospitalization numbers and stays should be interpreted carefully.

Further work in exploring the role air pollution has in the pathophysiology of sepsis could focus on markers of inflammation [28]. Inflammation has many players, including circulating cells and plasma proteins, vascular wall cells, and so on. A large scale study reporting an association between gaseous pollutants and C-reactive protein in diabetic patients [29]. This may explain that the associations were only significant in gaseous pollutants in our study. In our study, total sepsis estimates were based on both explicit and implicit with organ dysfunction codes. While implicit sepsis related to organ dysfunction might be developed gradually and is a chronic process. Short-term effect of air pollutants exposure may increase the sepsis hospitalizations by triggering acute episodes and exacerbations of implicit sepsis. The long-term effect of air pollutants on sepsis should be further studied in the future. We also explored the high-risk groups of different age and sex which were more vulnerable to air pollutant. These effect modification by age and sex was also observed in previous studies [7, 13]. Sepsis might be triggered to hospital admission due to diseases that are positively associated with short-term air pollution, making difference among age and sex groups.

The strengths of this analysis lie in the large number of sepsis cases. Besides, the ability to capture patients from over 6 typical Chinese cities allows for good external validity and generalizability. In addition, we evaluated a new causes of hospital admissions related to both gaseous pollutants and particulate matter that have rarely been studied. Finally, we further explored the effect of ambient air pollution on hospital stays for sepsis, which strengthens our findings.

This study also has some weaknesses. Firstly, we only evaluated the associations between air pollution and sepsis hospital admissions and stays, did not fully capture the corresponding costs associated with air pollution exposure. But we would estimate the economic burden of sepsis attribute to air pollution in the near future. Second, the lack of previous evidence requires further epidemiological studies and investigation of possible underlying mechanisms for this health effect. Finally, in our study, two-years research period is relatively short, and we need a longer time series for further research.

## Conclusion

This current study provide the evidence that exposure to NO_2_ and O_3_ may cause increased sepsis hospital admissions and hospital stays in Sichuan, China. Future research is needed to confirm these findings and explore potential mechanisms of the adverse effects of NO_2_ and O_3_ pollution in patients with sepsis.

## Supplementary Information

**Additional file1 Table S1.** Detailed ICD codes for the identification of sepsis. **Table S2.** Limit of pollutant concentration and Individual air quality index. **Table S3.** The city-specific percentage increase (estimates and 95% CI) in daily hospital admissions of sepsis associated with 10-μg/m^3^ increase in NO_2_ and O_3_ concentrations at lag day with the highest estimates of basic models for total populations. **Table S4.** Percentage increase (estimates and 95% CI) in daily hospital admissions of sepsis associated sepsis of 10-μg/m^3^ increase in PM_2.5_, PM_10_ and SO_2_ concentrations, and 1 mg/m^3^ increase in CO, stratified by age and sex.

## Data Availability

The datasets generated and/or analysed during the current study are not publicly available due to data sharing regulations but are available from the corresponding author on reasonable request.

## Abbreviations

PM_2.5_: Particulate matter with aerodynamic diameter ≤ 2.5 μm
PM_10_: Particulate matter with aerodynamic diameter ≤ 10 μm
NO_2_: Nitrogen dioxide
O_3_: Ozone
SO_2_: Sulphur dioxide
CO: Carbon monoxide
ICU: Intensive care unit
CD: Chengdu
MS: Meishan
MY: Mianyang
YB: Yibin
ZG: Zigong
LS: Yi autonomous prefecture
ICD-10: International classification of diseases 10th code
ICD-9: International classification of diseases 9th revision code
GAM: Generalized additive models
Df: Degrees of freedom
95% CI: 95% Confidence intervals
