# Supplementary material for: Sepsis-related hospital admissions and ambient air pollution: a time series analysis in 6 Chinese cities
Source: BMC Public Health. 2021 Jun 21;21:1182. doi: 10.1186/s12889-021-11220-x (PMC8218442; doi:10.1186/s12889-021-11220-x)
Supplement: Supplementary file 1 — Additional file1 Table S1. Detailed ICD codes for the identification of sepsis. Table S2. Limit of pollutant concentration and Individual air quality index. Table S3. The city-specific percentage increase (estimates and 95% CI) in daily hospital admissions of sepsis associated with 10-μg/m3 increase in NO2 and O3 concentrations at lag day with the highest estimates of basic models for total populations. Table S4. Percentage increase (estimates and 95% CI) in daily hospital admissions of sepsis associated sepsis of 10-μg/m3 increase in PM2.5, PM10 and SO2 concentrations, and 1 mg/m3 increase in CO, stratified by age and sex. [file 12889_2021_11220_MOESM1_ESM.docx]

Supplementary material

Sepsis-related hospital admissions and ambient air pollution: a time series analysis in 6 Chinese cities

**Yu Wang ^1,^** ^†^**, Zhen Liu ^2,^** ^†^**, Lian Yang^3,*^,** **Jiushun Zhou^4^ ,** **Jia Li^5^, HaiLun Liao^3^，XingJun Tian^6^**

1. Department of Anesthesiology, Union Hospital, Tongji Medical College, Huazhong University of Science and Technology, Wuhan 430022, China

2. Second Affiliated Hospital of Chengdu Medical College ·China National Nuclear Corporation 416 Hospital Chengdu, 610057, China

3. School of Public Health, Chengdu University of Traditional Chinese Medicine, Chengdu, 610075, China

4. Sichuan Center for Disease Control and Prevention, Chengdu, 610041, China

5. Management College, Chengdu University of Traditional Chinese Medicine， Chengdu, 610032, China

6. Sichuan Administration of TCM, Chengdu, 610016, China

^†^ These authors contributed equally to this work.

Corresponding authors: Lian Yang, PhD, School of Public Health, Chengdu University of Traditional Chinese Medicine, Chengdu, 610075, China

Email: yangliancd@163.com

**List of Tables in the Supplementary tables.**

**Table S1.** Detailed ICD codes for the identification of sepsis

**Table S2.** Limit of pollutant concentration and Individual air quality index

**Table S3.** The city-specific percentage increase (estimates and 95% CI) in daily hospital admissions of sepsis associated with 10-μg/m^3^ increase in NO_2_ and O_3_ concentrations at lag day with the highest estimates of basic models for total populations.

**Table S4.** Percentage increase (estimates and 95% CI) in daily hospital admissions of sepsis associated sepsis of 10-μg/m^3^ increase in PM_2.5_, PM_10_ and SO_2_ concentrations, and 1 mg/m3 increase in CO, stratified by age and sex.

| **Table S1.** Detailed ICD codes for the identification of sepsis | | |
| --- | --- | --- |
| **ICD Revision** | **Explicit sepsis** | **Implicit sepsis with Organ dysfunction** |
| **ICD-9** | 038-038.9, 090-097.9, 286.6, 635-639.9, 646.5-646.64, 658.4-658.93, 659.2-659.33, 670-670.9, 672-672.04, 674.1-674.34, 675-675.94, 771, 771.4-771.89, 800-801.99, 803-804.99, 905.0, 995.9-995.94 | 293-294, 295.4-295.45, 295.80-295.95, 296.82-298.9, 300.5-302.9, 306-307.0, 307.2-307.49, 307.6-307.7, 307.9, 310-310.1, 313-313.9, 316-316.9, 327-327.19, 327.3-327.8, 347-347.9, 584-584.9, 780-780.2, 780.93, 780.97, 797-797.9, 799.2-799.29, V11.0-V11.2, V11.4-V12.0, V17-V17.0, V40-V41.9, V79-V79.9 |
| **ICD-10** | A02.1-A02.9, A20.7-A20.9, A21.7-A21.9, A22.7-A22.9, A24.1-A24.9, A26.7-A26.9, A28.2-A28.9, A32.7-A32.9, A39.0, A39.4-A41.9, A42.7-A42.9, A50-A50.9, A54.86, B00.7-B00.9, B37.7-B37.9, N98.0, O03.0, O03.38, O03.5, O03.88, O04.5, O04.88, O07.38, O08.0, O08.83, O23-O23.93, O41.1-O41.93, O75.3, O85-O86.89, O88.3-O88.32, O91-O91.23, O98, O98.2-O98.93, P00.2, P22-P23.9, P29.12, P29.81, P35-P37, P37.1-P39.9, R65.2-R65.21, R68.13 | D65-D65.9, D69.5-D69.59, E87.2-E87.99, G93.4-G93.49, I46-I46.9, I95.1-I95.9, J80- J80.9, J95.2-J95.3, J96-J96.92, K72-K72.91, N00-N01.9, N17-N17.9, R09.02, R09.2, R40.0-R40.4, R41.82, R55-R55.0, R57-R57.9 |
| Abbreviations: ICD: International Classification of Diseases | | |
| Sepsis cases were identified using codes from the ICD 9th (ICD-9) and 10th (ICD-10) Revisions. | | |

| **Table S2.** Limit of pollutant concentration and Individual air quality index | | | | | |  |  |  |  |  |
| --- | --- | --- | --- | --- | --- | --- | --- | --- | --- | --- |
| Individual air quality index | Limit of pollutant concentration | | | | | | | | | |
|  | SO_2_  (24 hour average)  (µg/m^3^) | SO_2_  (1 hour average) (µg/m^3^) (1) | NO_2_  (24 hour average)  (µg/m^3^) | NO_2_  (1 hour average)  (µg/m^3^) (1) | PM_10_  (24 hour average)  (µg/m^3^) | CO (24 hour average)  (mg/m^3^) | CO  (1 hour average)  (mg/m^3^)(1) | O_3_  (1 hour average) (µg/m^3^) (1) | O_3_  (8 hour average)  (µg/m^3^) | PM_2.5_  (24 hour average)  (µg/m^3^) |
| 0 | 0 | 0 | 0 | 0 | 0 | 0 | 0 | 0 | 0 | 0 |
| 50 | 50 | 150 | 40 | 100 | 50 | 2 | 5 | 160 | 100 | 35 |
| 100 | 150 | 500 | 80 | 200 | 150 | 4 | 10 | 200 | 160 | 75 |
| 150 | 475 | 650 | 180 | 700 | 250 | 14 | 35 | 300 | 215 | 115 |
| 200 | 800 | 800 | 280 | 1200 | 350 | 24 | 60 | 400 | 265 | 150 |
| 300 | 1600 | (2) | 565 | 2340 | 420 | 36 | 90 | 800 | 800 | 250 |
| 400 | 2100 | (2) | 750 | 3090 | 500 | 48 | 120 | 1000 | (3) | 350 |
| 500 | 2620 | (2) | 940 | 3840 | 600 | 60 | 150 | 1200 | (3) | 500 |
| Abbreviation: IAQI: individual air quality index; PM_10_: particulate matter with aerodynamic diameter ≤10μm; PM_2.5_: particulate matter with aerodynamic diameter ≤2.5μm; SO_2_: sulfur dioxide; NO_2_:nitrogen dioxide; CO: carbon monoxide; O_3_: ozone; | | | | | | | | | | |
| Note: (1) 1 hour average concentration for real-time reporting only; | | | | |  |  |  |  |  |  |
| (2) When the 1-hour average concentration of sulfur dioxide is higher than 800µg/m^3^, the IAQI is calculated according to the 24-hour average concentration; | | | | | | | | | | |
| (3) When the 1-hour average concentration of ozone is higher than 800µg/m^3^, the IAQI is calculated according to the 24-hour average concentration.  (4) Air quality index level: 0 – 50, level I; 51 -100, level II; 101-150, level III; 151-200, level IV; 201-300, level V; 300+, level VI.  (From the Ministry of Ecology and Environment of the People's Republic of China: http://kjs.mee.gov.cn/hjbhbz/bzwb/jcffbz/201203/t20120302_224166.shtml) | | | | | | | | | | |

| **Table S3.** The city-specific percentage increase (estimates and 95% CI) in daily hospital admissions of sepsis associated with 10-μg/m^3^ increase in NO_2_ and O_3_ concentrations at lag day with the highest estimates of basic models for total populations. | | |
| --- | --- | --- |
| Cities | NO_2_ | O_3_ |
| CD | 2.57(-0.08,5.22) | 0.09(-0.46,0.63) |
| LS | 18.32(-13.05,49.70) | 1.79(-0.87,4.45) |
| MS | 3.22(-3.62,10.05) | 0.37(-0.58,1.32) |
| MY | 1.82(-4.57,8.20) | 1.51(0.27,2.75) |
| YB | 4.62(-2.38,11.62) | 0.59(-0.36,1.54) |
| ZG | 2.04(-5.07,9.15) | 1.19(0.17,2.21) |
| Abbreviations: CD, Chengdu; LS, Yi Autonomous Prefecture; MS, Meishan; MY, Mianyang; YB, Yibin; ZG, Zigong. | | |

| **Table S4.** Percentage increase (estimates and 95% CI) in daily hospital admissions of sepsis associated sepsis of 10-μg/m^3^ increase in PM_2.5_, PM_10_ and SO_2_ concentrations, and 1 mg/m3 increase in CO, stratified by age and sex. | | | | | |
| --- | --- | --- | --- | --- | --- |
| lag days | Age groups | | | Gender groups | |
|  | 0~13 | 14~65 | >65 | Male | Female |
| **PM_2.5_** |  |  |  |  |  |
| lag0 | -0.28 (-1.45, 0.90) | -0.66 (-1.57, 0.25) | -0.01 (-0.99, 0.98) | 0.06 (-0.86, 0.99) | -0.59 (-1.58, 0.40) |
| lag7 | 0.030 (-0.70, 0.76) | -0.09 (-0.73, 0.56) | -0.19 (-0.60, 0.97) | -0.06 (-0.68, 0.57) | 0.06 (-0.51, 0.62) |
| lag01 | -0.24 (-1.81, 1.34) | -0.52 (-1.74, 0.71) | -0.27 (-1.23, 0.69) | -0.03 (-0.99, 0.92) | -0.52 (-1.78, 0.76) |
| **PM_10_** |  |  |  |  |  |
| lag0 | 0.032 (-0.72, 0.79) | -0.43 (-1.07, 0.20) | 0.19 (-0.48, 0.87) | 0.16 (-0.47, 0.79) | -0.17 (-0.90, 0.56) |
| lag7 | 0.21 (-0.32, 0.73) | -0.01 (-0.47, 0.46) | 0.23 (-0.34, 0.79) | 0.10 (-0.35, 0.55) | 0.11 (-0.30, 0.52) |
| lag01 | 0.01 (-0.97, 0.98) | -0.30 (-1.13, 0.52) | -0.02 (-0.71, 0.67) | 0.08 (-0.60, 0.76) | -0.20 (-1.02, 0.63) |
| **SO_2_** |  |  |  |  |  |
| lag0 | 6.13 (-0.15, 12.40) | 2.86 (-4.82, 10.50) | 0.05 (-7.26, 7.36) | 4.68 (-0.64, 10.00) | 0.10 (-1.23, 1.43) |
| lag7 | 2.78 (-2.67, 8.24) | 1.77 (-6.21, 9.74) | -0.30 (-6.41, 5.81) | 1.20 (-3.48, 5.87) | 0.28 (-1.15, 1.70) |
| lag01 | 5.50 (-1.42, 12.40) | 1.51 (-9.69, 12.70) | -2.29 (-10.10, 5.52) | 3.74 (-2.47, 9.95) | 0.44 (-1.06, 1.93) |
| **CO** |  |  |  |  |  |
| lag0 | 12.50 (-3.48, 28.40) | 1.16 (-10.60, 12.90) | 10.80 (-7.30, 28.80) | 12.20 (-4.85, 29.30) | 0.89 (-9.63, 11.40) |
| lag7 | 6.46 (-4.65, 17.60) | -0.51 (-10.20, 9.22) | 1.94 (-10.00, 13.90) | -1.45 (-11.20, 8.28) | 4.06 (-4.54, 12.70) |
| lag01 | 18.30 (-2.52, 39.10) | 2.57 (-11.10, 16.30) | 12.70 (-4.00, 29.40) | 10.60 (-8.07, 29.40) | 4.01 (-3.41, 11.40) |
